# Coal tailings as a soil conditioner: evaluation of tailing properties and effect on tomato plants

**DOI:** 10.1007/s10725-022-00870-5

**Published:** 2022-07-21

**Authors:** Miing-Tiem Yong, Mohammad Babla, Shawan Karan, Utsab Katwal, Soheil Jahandari, Pushpinder Matta, Zhong-Hua Chen, Zhong Tao

**Affiliations:** 1 School of Science, Western Sydney University, Penrith, NSW 2751 Australia; 2 Technical Support Services and Mass Spectrometry Facility, Western Sydney University, Campbelltown, NSW 2560 Australia; 3 Centre for Infrastructure Engineering, Western Sydney University, Penrith, NSW 2751 Australia; 4 Hawkesbury Institute for the Environment, Western Sydney University, Penrith, NSW 2751 Australia

**Keywords:** Coal waste, Soil amendment, Mineral nutrients, Sustainable agriculture, Heavy metals, *Solanum lycopersicum* L.

## Abstract

The global coal industry yields a vast amount of tailings waste, and the utilisation of these tailings necessitates innovative efforts contributing to the United Nations Sustainable Development Goals. One of such novel initiatives is to reuse coal tailings (CT) safely, ecofriendly, and cost-effectively in agroecosystems as a soil conditioner to enhance the productivity of lands. This study aimed to evaluate the potential utilisation of coal tailings waste in the soil amelioration to improve plant performance. The physico–chemical characteristics of coal tailings from two Australian mining sites (CT1 and CT2) showed that the tailings samples are alkaline with loamy and loamy sand textures, respectively. The tailings have ~ 3% of macronutrients, high carbon (C), and low heavy metals and metalloids (As, Cd, Se, Cu, Zn, and Pb). The germination rate of tomato seeds was improved in the low-rate CT treatment. Greenhouse tomato plants exhibited an increase in leaf’s K, Ca, and Mg contents in CT1 and CT2 treatments. More importantly, the CT treatment-induced accumulation of heavy metals in plants was mostly insignificant in both CT treatments. Therefore, we highlight the potential application of coal tailings as a soil conditioner because of the beneficial effect of improved carbon and nutrients (N, P, K, Mg, and Ca) in tomato leaves. Further amendment of the coal tailings should focus on the adjustment of pH and the addition of other beneficial materials for the improvement of soil properties for crops in both the greenhouse and the field.

## Introduction

Globally, coal is a vital energy resource, which provides nearly 30% of the world’s energy consumption (Zhou et al. 2021). The energy supplied by coal consumption is expected to reduce to 24% of the total energy supply in 2040 (U.S. Energy Information Administration 2016), but future growth in coal consumption is expected to be mainly contributed from the developing countries in Asia (Clark et al. 2020). Coal production is one of the main industries in Australia, contributing to the Australian economy and national energy supplies. The Australian coal-fired power plants generated over 60% of national electricity production in 2019 (World Nuclear Association 2019). Meanwhile, the coal exports accounted for AU$37 billion of export revenue in 2021 and are expected to reach over AU$50 billion after the Covid-19 crisis (Australian Government 2021).

Processing raw coal into saleable coal inevitably generates large amounts of wastes that adversely affect the environment and increase greenhouse gas emissions. Coal processing typically produces around 30% of coal wastes from raw coal, including about 25% coarse coal gangue and 5% coal tailings (Adiansyah et al. 2017; Mohammadi et al. 2020). In addition, coal combustion generates other major wastes—more than 1000 Mts of fly ash and bottom ash from coal-fired power plants worldwide (Han et al. 2021; Zhou et al. 2021). Most of the coal wastes were either landfilled or stockpiled for future use (Dellantonio et al. 2010; Mohammadi et al. 2020). Therefore, it has drawn increasing research interests to find alternative uses of coal wastes to lessen the pressure on the storage facilities and to eliminate the hazards to the environment and human health. While significant research progress has been made in utilising fly ash, bottom ash, and coal gangue (Zhou et al. 2021), there is limited research work into the utilisation of coal tailings (CT). In China, it was reported that only a small amount of coal tailings has been used in manufacturing construction materials (Liu 2010). Therefore, there is an urgent need to conduct comprehensive research to transform CT into sustainable products as an alternative coal waste disposal solution (Babla et al. 2022).

Coal wastes usually have a good amount of carbon, macronutrients, and micronutrients (Haynes 2009). The total carbon contents in coal wastes were frequently reported to be between 20 and 50%. Brown coal waste, fly ash, bottom ash, and gauge were reported to be used as soil conditioning materials that can increase soil organic carbon content, which is directly correlated to soil organic matter (Amoah-Antwi et al. 2020; Zhou et al. 2021). Organic matter has an important impact on soil physical structure, microbial activities, and nutrient retention capability for crop production. The beneficial effects of soil organic matter are indirect and reflected in the long-term by improving the overall properties of soil (Azadi et al. 2019). In addition, coal waste with high carbonate content has a strong liming effect of ameliorating acidic soil (Manoharan et al. 2010). Effective amelioration of alkaline soil was also reported by using coal wastes with a high sulphate content (Chen et al. 2013). Budak et al. (2020) reported that Ca and Al contents in soil increased after CT treatment alone, while a combined treatment of CT and mycorrhizal fungi improved the germination rate, shoot length, root number, root length, and N and P contents in the root and shoot in perennial ryegrass and Kentucky bluegrass. Tremain et al. (2014) pointed out the potential benefit of applying charcoaled coal tailings to soil to improve soil physical structure of both sandy and clayey soils.

However, the possible presence of an excessive amount of trace elements in coal wastes can result in pollution to soil, water, plants, and food, affecting humans and animals (Yunusa et al. 2012; Diao et al. 2018; Fu et al. 2019; Han et al. 2021; Wang et al. 2021). Moreover, Singh et al. (2016) reported that the heavy metal content in crops was correlated to the heavy metal content in the soil. The coal gauge treatment led to an increased heavy metal content in soil with increasing times of application (Li et al. 2011; Zhang et al. 2018). Further investigation is still needed on the ameliorative effect of CT and possible heavy metal contamination to agricultural soil.

In this study, we evaluated the potential application of CT waste as a soil conditioner for sustainable agriculture and compared heavy metals content in tomato plants receiving different CT treatments. We hypothesised that CT treatment at a low application rate could improve tomato crop growth without heavy metal contamination in plants. Firstly, the physical and chemical characterisation were conducted on coal tailings collected from two mining sites in Queensland and New South Wales, Australia, respectively. The measured properties include particle size distribution, pH, electric conductivity (EC), and chemical composition. Then the effect of CT on plant growth was evaluated by applying CT to tomato plants in comparison with the control in the greenhouse. Measurements of plant growth and photosynthesis were conducted on the tomato plants. The feasibility of using CT as a carbon and nutrient source to improve soil fertility was evaluated.

## Materials and methods

### Characterisation of coal tailings

Coal tailings slurry was collected from two mining sites in Australia, and the corresponding tailings samples were designated as CT1 and CT2, respectively. The tailings slurry was then oven-dried at 105 °C for 4 days to remove moisture. The dried samples were hand-ground and sieved through 2 mm sieves. The pH and EC were measured according to Tirez et al. (2014). The sample solution was prepared by mixing one part of sample with five parts of distilled water (w/w). The sample solution was mechanically shaken overnight. EC and pH were measured after half-hour of stabilisation. Particle size analysis of the tailings was conducted according to the Australian standards AS 1289.3.6.1 (Standards Australia 2009) and AS 1289.3.6.3 (Standards Australia 2020). The samples for measuring the total carbon and nitrogen contents were prepared by fine grinding using a ball miller MM 400 (Retsch, Verder Scientific, Germany) at a frequency of 30 Hz for 2 min. The total carbon (C) and nitrogen (N) contents in the CT were quantified using a LECO TruMac CN analyser (LECO Corporation, USA). Based on the Dumas method (Wang et al. 2015b), 200 mg of dried and ground samples were combusted at 1100 °C for each measurement.

### Element analysis of coal tailings and tomato leaves

Contents of key elements (K, P, Ca, Mg, Al, Fe, Mn, Zn, Cu, Cd, Pb, As, and Se) in the CT were measured using Inductively Coupled Plasma Mass Spectrometry (ICP-MS, Perkinelmer NexION 5000 Multi-Quadrupole) according to EPA (2007). Sieved samples were milled using a Retsch ball miller MM 400 at a frequency of 30 Hz for 2 min. A 150 mg of sample was digested in ultrapure concentrated nitric acid (4 ml) and hydrogen peroxide (2 ml) mixture. The digestion was carried out in a closed vessel system (Speedwave 4 microwave digester) at 220 °C, > 25 bars pressure, for 2 h. The digested sample was then diluted into 100 ml and filtered using 22 µm filters. The elemental content in the sample solution was measured using ICP-MS within the detection range from 0.001 to 1 mg L^−1^. The sample solution was further diluted 10 and 100 times with 2% HNO_3_ solution for the abundant elements.

Macronutrient (K, P, Ca), micronutrient (Mg, Fe, Mn, Zn, Cu, and Se), and heavy metal (Al, Cd, Pb, and As) contents in tomato leaves were quantified using ICP-MS according to O’Carrigan et al. (2014). The leave samples were powdered using a Retsch ball miller MM 400 at 1800 RPM for 2 min. For elemental measurements using ICP-MS, a 200 mg sample was digested in a mixture of 4 ml ultrapure concentrated nitric acid and 1 ml hydrogen peroxide. The digestion was carried out at 100 °C on a hot plate until the sample was completely digested and the acid solution was evaporated. The digested sample was diluted to 50 ml and filtered using 22 µm filters. The elemental contents in the sample solution were measured using ICP-MS within the detection range from 0.001 to 1 mg/L. The sample solution was further diluted 10 and 100 times with 2% HNO_3_ solution for abundant elements measurements.

### Plant materials and growth condition

The short-term effect of coal tailings was performed in the growth chamber [26 °C, RH 60%, 200 μmol m^−2^ s^−1^ photosynthetically active radiation (PAR)]. Tomato (*Solanum lycopersicum* L.) *cv*. Black Krim seeds were sown and germinated in a 2 L pot filled with potting mix. 0–20% CT (CT1 or CT2) treatments were premixed into the potting mix based on the air-dried density of potting mix of 0.37 kg L^−1^. The germination rate was counted at 10 days after sowing. Aboveground tissue was harvested for biomass measurement at 3 weeks old.

For the greenhouse trial, evaluation of CT1 and CT2 was conducted in two separate batches as materials were received separately. Trials of CT1 and CT2 were conducted in August 2020 and November 2020, respectively. Firstly, tomato seeds were germinated in the seedling-raising potting mix. Healthy and uniform tomato seedlings were grown for 4 weeks inside a growth chamber with full-strength Hoagland nutrient solution weekly. To study the effect of coal tailings, 0–20% CT (dry weight-based, W/W) were mixed into the potting mix. Four weeks old seedlings were then transplanted into 3-L pots with potting mix. The plants were grown in the greenhouse for 11 weeks at around 300 μmol m^−2^ s^−1^ of PAR throughout the entire experiment. The general growth conditions were 26 ± 2 °C (60% RH) during the day and 22 ± 2 °C (70% RH) during the night under a 16/8 h light/dark cycle. The plants were well-watered and fertilised at half strength with a commercial fast release fertiliser (Hortico Aquasol, Yates, Victoria, Australia) every fortnight during the two experimental trials.

### Plant growth and biomass

Plant growth was determined by measuring the plant height and the number of fully expanded leaves fortnightly. At the end of the greenhouse trial, all the tomato plants were harvested to determine the above ground fresh weight and then dried in an oven at 70 °C for one week to determine their dry weight.

### Gas exchange measurements

A portable LI-6400XT infrared gas analyser (Li-Cor Inc., Lincoln, NE, USA) was used to conduct the instantaneous steady-state leaf gas exchange measurements from fully expanded top canopy leaves, according to (Babla et al. 2020). Net CO_2_ assimilation (*A*, µmol m^−2^ s^−1^), stomatal conductance (*g*_*s*_, mol m^−2^ s^−1^), water use efficiency (*WUE*), defined as the ratio of *A* to *g*_*s*_, intercellular CO_2_ concentration (*C*_*i*_, µmol mol^−1^), and transpiration (*T*_*r*_, mmol m^−2^ s^−1^) were determined fortnightly. The conditions in the measuring chamber were controlled at a flow rate of 500 mol s^−1^, at saturating PAR of 1500 µmol m^−2^ s^−1^, 400 μmol mol^−1^ CO_2_, 25 °C leaf temperature and a relative humidity of 60–70%.

### Statistical analysis

Analysis of Variance (ANOVA), Student *t*-test, and Duncan’s multiple range tests (DMRTs) were performed using IBM SPSS Statistics (IBM Corp. Version 24, USA). Figures and tables were generated using SigmaPlot (Systat Software Inc., Version 14.5, USA) and Microsoft Excel (Microsoft Inc., Office16, USA).

## Test results

### Properties and elemental characterisation of coal tailings

Compared to CT2, CT1 had higher clay (20% vs 15%) and silt (30% vs 15%) contents and lower sand content (50% vs 70%) (Table 1). CT1 and CT2 were classified as loam soil and sandy loam soil, respectively, according to the soil texture classification chart (Skaggs et al. 2001). Both CT1 and CT2 were slightly alkaline (pH: 8–9) and non-saline (EC: < 1 dS m^−1^) (Table 2).

**Table 1:** Particle size distribution of tailings from two Australian coal mining sites

Sample	Clay (< 0.002 mm) (%)	Silt (0.002–0.05 mm) (%)	Sand (> 0.05 mm) (%)	Texture
CT1	~ 20	~ 30	50	Loam
CT2	~ 15	~ 15	70	Sandy loam

**Table 2:** Chemical properties of tailings from two Australian coal mining sites

	CT1	CT2
pH & EC		
pH	8.7 ± 0.3	8.1 ± 0.03
EC	0.91 ± 0.01	0.61 ± 0.02**
Elemental content (g kg^−1^)		
N	6.40 ± 0.04	11.19 ± 0.14**
P	0.81 ± 0.12	1.01 ± 0.06
K	21.29 ± 1.92	23.92 ± 0.60
C	243.59 ± 0.88	476.49 ± 0.60**
Al	111.75 ± 13.54	120.26 ± 4.92
Ca	7.63 ± 1.51	7.47 ± 0.40
Fe	34.89 ± 3.47	34.85 ± 1.24
Mg	7.26 ± 0.66	8.60 ± 0.21

The data are mean values (± SE, n = 4)

**Indicates significant Student *t*-test between CT1 and CT2 at *P* < 0.01

The elemental analysis indicated that C is the most abundant component in both CT1 and CT2 (Table 2). The total C content of 476.5 g kg^−1^ in CT2 was significantly (*t*-test, *P* < 0.01) higher than the corresponding value of 243.6 g kg^−1^ in CT1. The next most abundant components in both CT1 and CT2 were Al (111.8–120.3 g kg^−1^) and Fe (~ 35 g kg^−1^), which can be harmful to plant growth when their presence in the environment is in exchangeable forms and large quantities. Important plant macronutrients Mg, Ca, N, P, and K in both CT1 and CT2 were in the range between 0.81 and 23.9 g kg^−1^. The contents of macronutrients in CT1 were mostly lower than those in CT2. Compared with the contents of other macronutrients, the P content was the lowest in both CT1 (0.81 g kg^−1^) and CT2 (1.0 g kg^−1^), followed by the Mg and Ca contents (7.26–8.60 g kg^−1^). The N content in CT2 (11.2 g kg^−1^) was significantly (*t*-test, *P* < 0.01) higher than that in CT1 (6.4 g kg^−1^). The K content was the highest among the macronutrients in CT1 (21.3 g kg^−1^) and CT2 (23.9 g kg^−1^).

The heavy metal contents in both CT1 and CT2 were overall below the safe levels specified by the Australian standard AS 4454-2012 (Table 3). The metalloid As content in both CT samples was close to the allowed limits for soil conditioner products. It is followed by the Se contents ranging between 45 and 65% of the standard limit of 5 mg/kg and the Zn contents ranging between 25 and 40% of the 300 mg/kg limit. The remaining elements were below 40% of the allowed limits.

**Table 3:** Heavy metal contents in tailings from two Australian coal mining sites

Element (mg kg^−1^)	CT1	CT2	AS 4454-2012
Cu	39.95 ± 8.03	34.55 ± 12.44	150
Zn	117.51 ± 28.24	77.49 ± 7.38	300
As	13.48 ± 1.44	15.59 ± 2.97	20
Cr	27.79 ± 4.77	31.61 ± 2.70	100
Pb	19.59 ± 2.35	20.98 ± 2.32	150
Se	2.36 ± 0.30	3.21 ± 0.10*	5
Cd	0.17 ± 0.02	0.22 ± 0.02	1

The data are mean values (± SE, n = 4)

*Indicates significant Student *t*-test between CT1 and CT2 at *P* < 0.05

### Tomato growth at early growth and vegetative stages

**Fig. 1:**
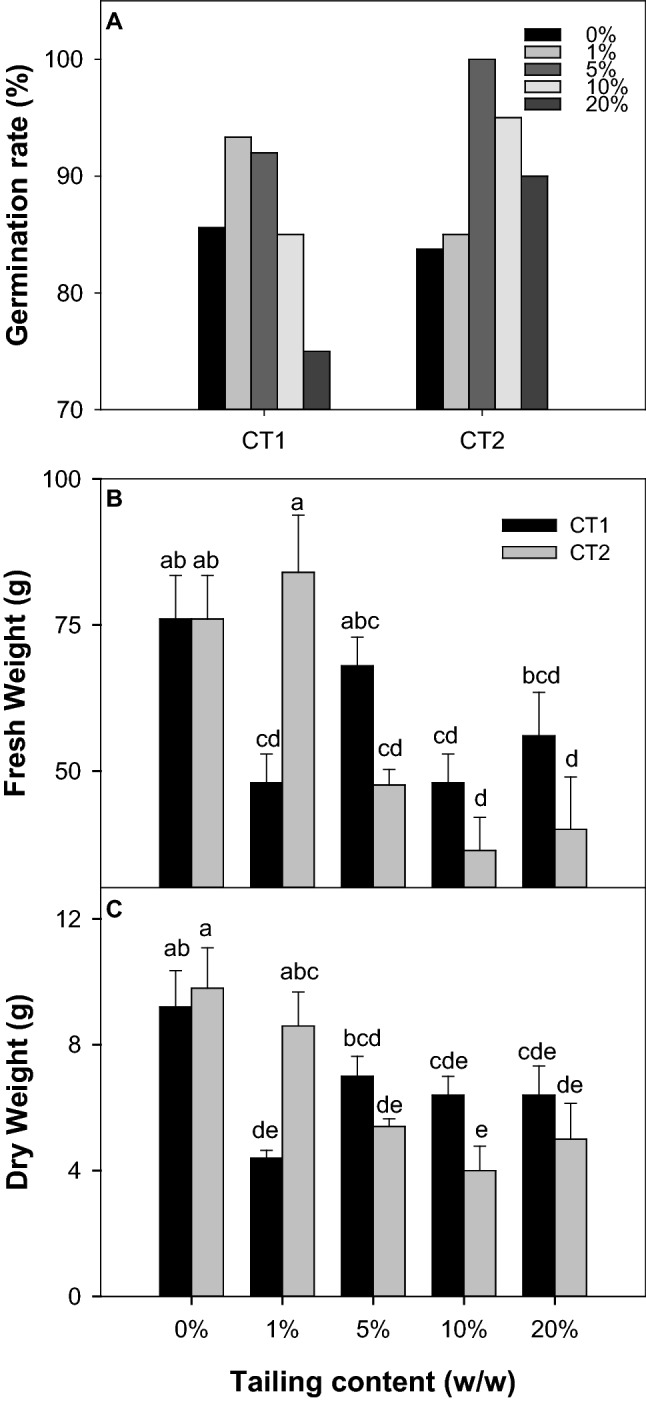
Effect of coal tailings on seed germination and growth at the seedling stage of tomato. **A** Germination rates of all seeds were measured after 10 days of germination. **B** Fresh weight, and **C** Dry weight were measured after 3 weeks of growth. Different lowercase letters indicate significant differences at *P* < 0.05

In response to CT treatments, germination of seeds sown in potting mix with CT1 treatments was the highest at low-rate treatments (1% and 5%) (Fig. 1A). There is no difference observed between the control and 10% treatment, but the germination rate was significantly reduced in the 20% treatment. Overall, all CT2 treatments improved germination rates of tomato seeds. Similar to CT1 treatments, the germination rate was higher at lower rates of treatment. After 3 weeks of growth (Fig. 1B, C), the overall growth of tomato plants was adversely affected by the CT treatment (ANOVA, Treatment effect, *P* < 0.001). A significant interactive effect (ANOVA, Treatment × Time effect, *P* = 0.001) suggested that the difference between samples (CT1 & CT2) became significant when a higher application rate of CT was used. While CT2 did not significantly affect the early plant growth at a low concentration treatment (1%), the adverse effect of CT2 became evident at a higher application rate (5–20%) in comparison with the CT1 treatment. It was also shown in Duncan’s Multiple Range Test result, where the biomass of plants with 10% and 20% treatments fell within the lowest-ranked groups (Group d and e).

**Fig. 2:**
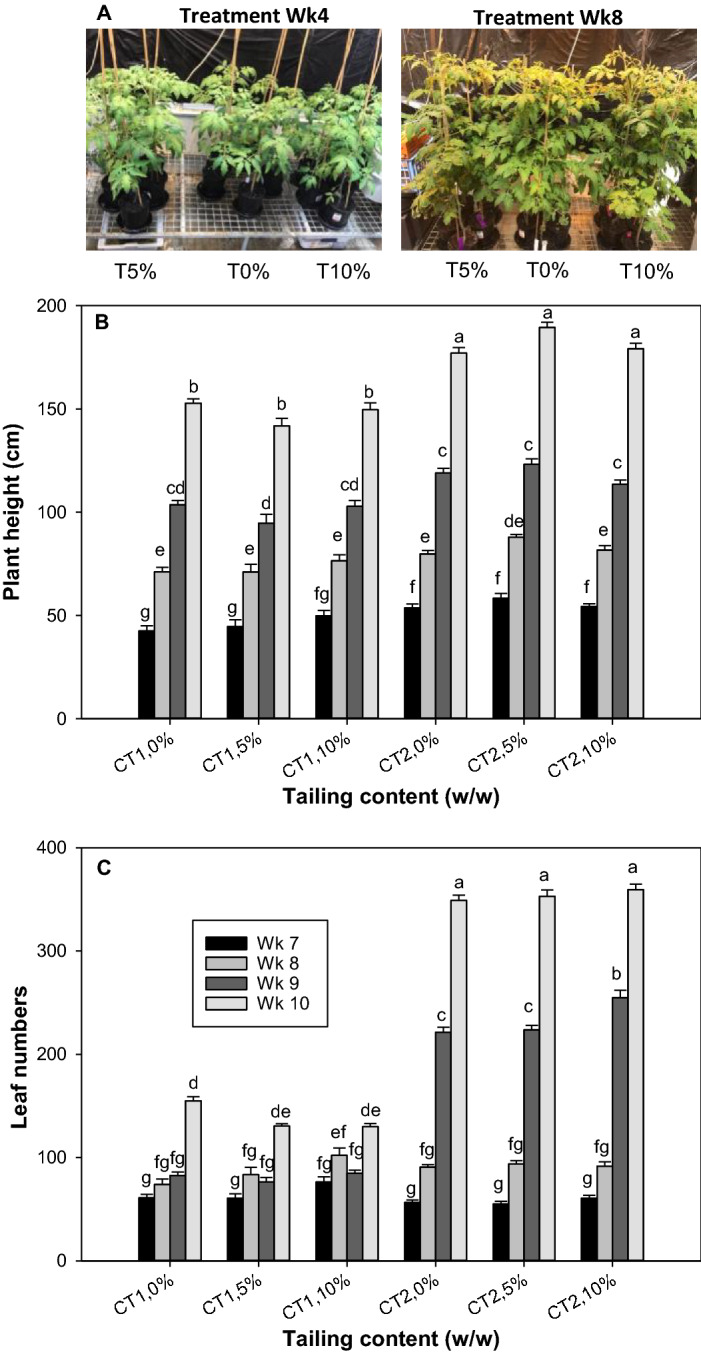
Long-term effect of coal tailings from the two mining sites on the morphological traits of tomato. The data are mean values ± SE (n = 5) of **A** appearance of plant after 4 & 8 weeks of CT treatment (CT2), **B** plant height, and **C** leaf numbers. Plant height and leaf numbers were measured weekly from Week 6 to Week 10 after transfer to greenhouse. Different lowercase letters indicate significant differences at *P* < 0.05

**Fig. 3:**
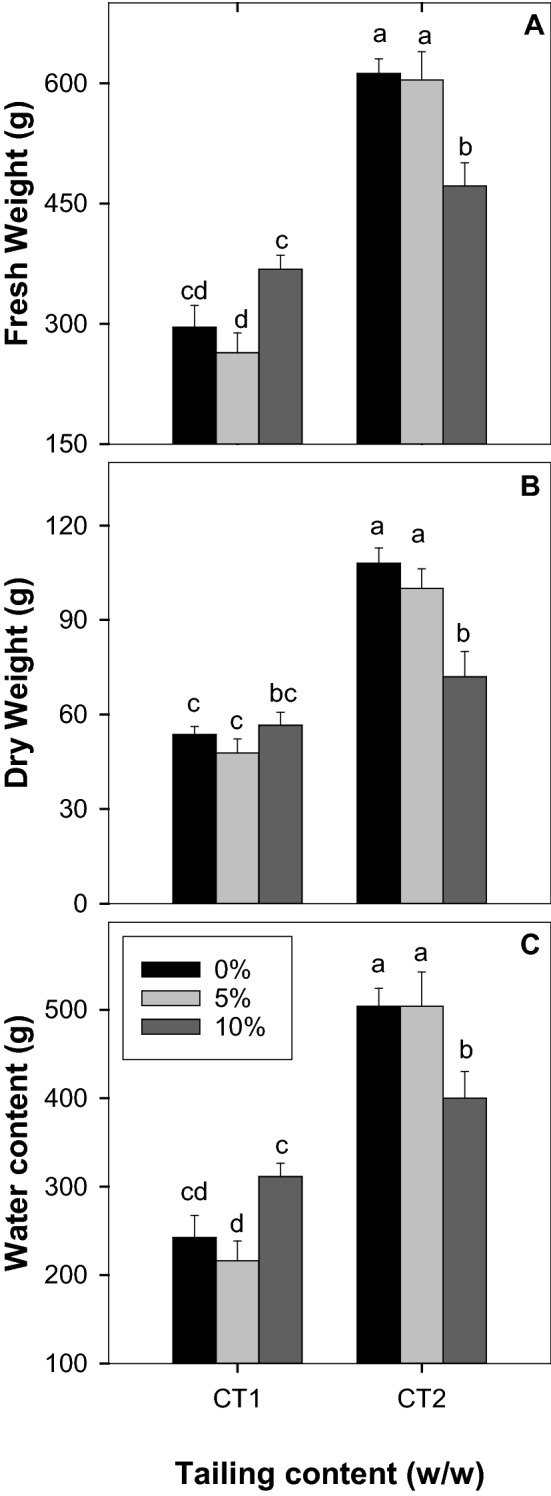
Long-term effect of coal tailings from the two mining sites on the biomass of tomato plant. The data are mean values ± SE (n = 5) of **A** fresh weight, **B** dry weight and **C** water content of tomato plants after 12 weeks of treatment. Different lowercase letters indicate significant differences at *P* < 0.05

In terms of the long-term effect of the CT treatment in the greenhouse, the influence of CT on the plant height and leaf numbers is not significant (Fig. 2B, C, ANOVA, treatment effect, *P* > 0.05). The appearance of the plants after 4 and 8 weeks of CT treatment is shown in Fig. 2A. Nonetheless, plants with CT1 treatment showed an insignificant reduction in biomass at a 5% application rate followed by a slight increase of biomass at a 10% application rate in contrast to the control without CT treatment (Fig. 3A–C). Compared to the control, plant biomass was not affected after a long-term 5% CT2 treatment but was significantly reduced after a long-term 10% CT2 treatment (Fig. 3A, C). The results suggested that the raw CT treatment at a low percentage (e.g., 5%) did not reduce the plant growth.

### Leaf gas exchange

Leaf gas exchange and photosynthesis are key indicators of plant health and yield potential (Babla et al. 2020). Table 4 shows the comparisons of five gas exchange parameters (*A*, *gs*, *WUE*, *VpdL*, and *Trmmol*) between plants under different rates of CT1 and CT2 treatments from Week 7 to Week 11 of treatment in the greenhouse. A two-way ANOVA analysis (Interactive effects, *P* < 0.01) indicates that the CT1 treatment led to a gradual increase in *g*_*s*_ and *Trmmol* and reduced *VpdL* of the leaves. The results indicate that the plants with CT1 treatment had higher water consumption; however, the CT treatment-induced water consumption did not increase the net CO_2_ assimilation rate. Leaf gas exchange measurements of plants did not show a significant difference between the control and plants with CT2 treatments apart from the significant effect of time (week) of the CT2 treatment (Table 4). The treatment effect and interactive effect of treatment × time of five gas exchange parameters were overall insignificant. The results showed that the effect of CT2 treatment on the leaf gas exchange properties is insignificant.

**Table 4:** Effect of coal tailings treatments on the photosynthetic traits of tomato leaves

Rate (w/w)	Wk	*A*		*gs*		*WUE*		*VpdL*		*Trmmol*	
		CT1	CT2	CT1	CT2	CT1	CT2	CT1	CT2	CT1	CT2
0%	7	15.7 ± 1.13^a^	15.5 ± 0.58^ ns^	0.22 ± 0.02^bcd^	0.53 ± 0.07^a^	75.9 ± 13.5^abc^	31.4 ± 3.7^c^	2.34 ± 0.07^ab^	1.6 ± 0.07^c^	4.8 ± 0.43^b^	7.0 ± 0.5^a^
	9	15.0 ± 0.37^ab^	15.4 ± 0.54^ ns^	0.28 ± 0.01^b^	0.31 ± 0.02^ab^	54.7 ± 2.2^ cd^	48.9 ± 2.4^bc^	1.97 ± 0.02^c^	1.9 ± 0.04^abc^	5.1 ± 0.18^b^	5.6 ± 0.2^ab^
	11	12.7 ± 0.59^c^	14.5 ± 1.56^ ns^	0.26 ± 0.03^bc^	0.3 ± 0.12^ab^	51.6 ± 6.1^ cd^	74.0 ± 17.0^a^	1.99 ± 0.09^c^	2.0 ± 0.19^ab^	4.7 ± 0.38^b^	4.6 ± 1.22^b^
5%	7	13.7 ± 1.55^ab^	16.4 ± 0.48^ ns^	0.19 ± 0.02^ cd^	0.46 ± 0.08^ab^	81.7 ± 14.9^ab^	41.3 ± 7.5^bc^	2.50 ± 0.09^a^	1.7 ± 0.09^bc^	4.3 ± 0.48^b^	6.3 ± 0.69^ab^
	9	14.6 ± 0.38^ab^	13.5 ± 0.46^ ns^	0.25 ± 0.01^abc^	0.37 ± 0.03^ab^	60.2 ± 2.5^bc^	37.8 ± 4.2^bc^	2.04 ± 0.02^c^	1.8 ± 0.07^abc^	4.8 ± 0.15^b^	6.2 ± 0.32^ab^
	11	14.2 ± 0.65^ab^	14.8 ± 0.94^ ns^	0.43 ± 0.02^a^	0.25 ± 0.03^b^	33.0 ± 1.6^d^	61.3 ± 7.6^ab^	1.70 ± 0.03^d^	2.1 ± 0.09^a^	6.5 ± 0.21^a^	4.8 ± 0.47^b^
10%	7	14.9 ± 0.99^ab^	15.6 ± 0.92^ ns^	0.18 ± 0.02^d^	0.46 ± 0.05^ab^	93.5 ± 9.6^a^	34.8 ± 1.9^bc^	2.51 ± 0.07^a^	1.6 ± 0.06^c^	4.1 ± 0.37^b^	6.4 ± 0.36^ab^
	9	13.4 ± 0.53^ab^	13.5 ± 0.40^ ns^	0.21 ± 0.01^abc^	0.42 ± 0.06^ab^	66.0 ± 5.2^bc^	35.5 ± 6.5^bc^	2.17 ± 0.04^bc^	1.8 ± 0.09^abc^	4.4 ± 0.16^b^	6.4 ± 0.62^ab^
	11	14.8 ± 0.52^ab^	14.9 ± 1.45^ ns^	0.45 ± 0.03^a^	0.39 ± 0.09^ab^	33.4 ± 2.5^d^	47.3 ± 10.5^bc^	1.68 ± 0.04^d^	1.8 ± 0.15^abc^	6.6 ± 0.31^a^	5.7 ± 0.85^ab^
Treatment		ns	ns	ns	ns	ns	ns	ns	ns	ns	ns
Time		**	**	**	**	**	**	**	**	**	**
Interaction		ns	ns	**	ns	ns	ns	**	ns	**	ns

The data are mean values (± SE, n = 5). Duncan Multiple Range Test’s comparison was conducted separately for each parameter of each CT treatment. Different superscripted lowercase letters indicate significant differences at *P* < 0.05 for each Duncan group and superscripted ns indicates no group was formed at *P* < 0.05

** and ns indicate significant and insignificant ANOVA result at *P* < 0.01 and *P* > 0.05, respectively

### Macronutrients, micronutrients, and heavy metals in leaves

Tomato plants under coal tailings treatments generally had higher nutrient (e.g., K, P, Ca, and Mg) contents in the leaves. The content of these macronutrients in the leaves of plants with 5% CT2 treatment was slightly higher than that of the control. But the plants with 10% CT2 treatment showed significantly (*t*-test, *P* < 0.01) higher nutrients accumulated in the leaves. For CT1 treatments, there was no significant (*t*-test, *P* > 0.05) difference in the contents of K and P between the control and the CT treated plants with 5% and 10% CT (Table 5).

**Table 5:** Long-term effect of coal tailings on the nutrient contents in tomato leaves

Nutrient (g kg^−1^)	Sample	Treatment 0%	Treatment 5%	Treatment 10%
K	CT1	34.15 ± 6.15	34.56 ± 5.93	36.99 ± 2.07
	CT2	18.19 ± 1.34	20.5 ± 3.07	32.05 ± 1.64**
P	CT1	4.07 ± 0.57	4.17 ± 0.25	4.70 ± 0.10
	CT2	3.06 ± 0.29	3.3 ± 0.63	5.11 ± 0.18**
Ca	CT1	5.19 ± 2.84	12.92 ± 0.14	12.05 ± 0.50
	CT2	7.52 ± 0.80	8.27 ± 0.87	12.26 ± 0.64**
Mg	CT1	4.19 ± 1.33	7.25 ± 0.32	6.82 ± 0.37
	CT2	5.34 ± 0.63	6.07 ± 0.94	8.60 ± 0.10**
Fe	CT1	0.06 ± 0.00	0.06 ± 0.00	0.08 ± 0.00
	CT2	0.12 ± 0.02	0.2 ± 0.02	0.13 ± 0.01
Al	CT1	0.03 ± 0.00	0.02 ± 0.00	0.03 ± 0.00
	CT2	0.02 ± 0.00	0.02 ± 0.00	0.03 ± 0.00

The data are mean values (± SE, n = 4)

**Indicates significant Student *t*-test analysis at *P* < 0.01 compared to the control

The Fe and Al contents in plants receiving CT treatments were negligible (< 0.2 g kg^−1^) (Table 5), indicating that the abundant Fe and Al in CT samples were mainly present in bound forms that are unavailable for plant uptake (Table 2). The leaf samples of plants with 10% CT1 treatment showed significantly higher As accumulation (*t*-test, *P* < 0.05) in comparison to those of the control and plants with 5% CT1 treatment (Table 6). Furthermore, the Cd content was significantly increased in the leaves of samples treated with both 5% and 10% CT1. Given the higher Cd content in CT2 samples (Table 3), it is surprising to observe the higher accumulation of Cd in CT1 treated plants than in CT2 treated plants. It indicates that other properties of CT, such as types of tailings and pH, may have affected the heavy metal uptake of tomato plants.

**Table 6:** Long-term effect of coal tailings on the heavy metal accumulation in tomato leaves

Element (mg kg^−1^)	Sample	Treatment 0%	Treatment 5%	Treatment 10%
As	CT1	0.04 ± 0.01	0.05 ± 0.01	0.11 ± 0.01**
	CT2	0.04 ± 0.00	0.05 ± 0.01	0.06 ± 0.01*
Cd	CT1	0.04 ± 0.01	0.07 ± 0.00*	0.10 ± 0.01**
	CT2	0.06 ± 0.03	0.05 ± 0.01	0.04 ± 0.00
Cu	CT1	6.8 ± 1.39	5.77 ± 0.27	6.17 ± 0.28
	CT2	7.34 ± 0.45	7.23 ± 0.95	9.8 ± 0.46*
Pd	CT1	0.53 ± 0.02	0.92 ± 0.26	0.75 ± 0.11
	CT2	1.00 ± 0.16	0.97 ± 0.19	0.64 ± 0.1
Se	CT1	0.11 ± 0.02	0.14 ± 0.01	0.18 ± 0.01
	CT2	0.11 ± 0.01	0.12 ± 0.01	0.15 ± 0.01
Zn	CT1	15.52 ± 1.78	14.12 ± 1.2	20.62 ± 2.32
	CT2	12.99 ± 1.71	17.55 ± 3.42	16.65 ± 1.45

The data are mean values (± SE, n = 4)

* and **Indicate significant Student *t*-test analyses at *P* < 0.05 and *P* < 0.01, respectively compared to the control

## Discussion

### Coal tailings improve nutrient uptake without heavy metals accumulation in plants

Poor management and monitoring of coal wastes can lead to disastrous events, such as dam failure, toxic contamination, and explosion due to self-ignition (Hatje et al. 2017; Park et al. 2019). Alternatively, coal tailings can potentially be used to remediate soil fertility for crops as the tailings might contain available nutrients and low heavy metal contents. Meanwhile, CT generally contains huge amounts of organic carbon and Ca/Mg carbonate compounds that may act as an absorbent to reduce heavy metal pollution and a liming agent to remediate acidic soil (Spain and Tibbett 2012; Wang et al. 2015a). However, further research should be conducted on the potential use of CT for soil amelioration for agricultural application.

From this study, we found that CT from both sites contain high amounts of plant macronutrients such as K, Ca, and Mg and some N and P. However, the application rate of 5% or above will be too high if we convert the rate to tonne ha^−1^ for field applications. We estimated that 1% of coal tailings in the potting mix with an air-dried density of 370 g L^−1^ is equivalent to 11 tonnes ha^−1^ based on a topsoil depth of 30 cm. Therefore, the application of 5% or 10% CT will introduce massive amounts of various elements to the soil and plants [e.g., 5% CT application rate (tonnes ha^−1^): C, 13–27; K, 1; N, 0.4–0.6; P, 0.05; Ca, 0.4; and Mg, 0.5). The supplements of 5% CT in our study were higher than the required nutrients for plants and higher than the supplements applied in many other studies, except P (Ward 1964; Kanai et al. 2011; Cheng et al. 2021). Surprisingly, our results indicated that these nutrients in tomato leaves only slightly increased with 5% CT treatments and significantly increased with 10% treatments. Interestingly, the plant biomass was not correlated to the nutrient content in leaves. In both CT treatments, the 5% treatments did not show a significant effect on the biomass of tomatoes in the long-term. The 10% CT1 treatment only slightly increased biomass in the long-term without a significant impact on the nutrient content, whereas 10% CT2 treatment reduced biomass but significantly increased the nutrient content. Phytotoxicity due to excessive fertilisation or nutrient deficiency can be excluded as the nutrient contents in leaves were within the normal ranges for tomato reported elsewhere (Juan et al. 2007; Suzuki et al. 2015).

By measuring the heavy metal content in leaves, CT1 treated samples surprisingly had higher As and Cd accumulation than CT2 treated samples, while the contents of heavy metals in CT1 treated samples were slightly higher than those in CT2 treated samples (Tables 2 and 6). However, no biomass reduction was observed in CT1 treated plants, and only a small reduction was found in 10% CT2 treated plants. Higher heavy metals in plants with CT1 treatment may be related to the high total carbon content in CT2, which consists of organic carbon and inorganic carbon (Ontl and Schulte 2012). Organic carbon content is directly related to the soil organic matter content in the soil, and inorganic carbon generally refers to the carbon in carbonate form. Both organic and inorganic carbon have been reported for their outstanding ability to remediate polluted soil via locking down different plant pollutants, such as heavy metals and pesticides (Skłodowski et al. 2006; Ouhadi et al. 2010; Placek et al. 2017; Carpio et al. 2021). Furthermore, the content of plant extractable heavy metals was found to be affected by the particle size distribution of soil (Clemente et al. 2020; Carpio et al. 2021). Small soil particles were found to have higher plant extractable Zn and As contents. We found that the clay and silt contents in CT1 are much higher than those in CT2 (Table 1), thus leading to higher extractable heavy metals in CT1 for uptake by the tomato plants. The stomatal conductance of plants was maintained or increased in response to CT treatment, which may indicate that an increased accumulation of low levels of heavy metals in the tomato plants were not sufficient to trigger phytotoxicity (Białowiec et al. 2019).

### Potential use of coal tailings in the amendment of acidic soils by modifying their pH value

Across the world, acidic soils limit crop production because of reduced accessibility of soil nutrients and increased possibility of metal toxicity, such as Al and Mn (Bojórquez-Quintal et al. 2017). Many agricultural activities, such as the application of fertilisers (e.g., urea, sulphur) and organic material decomposition, were reported to increase soil acidity. CT could be valuable to agriculture and environmental restoration of acid soils as the soil amendment is of global interest (Feng et al. 2020; Liu et al. 2021). Our study demonstrates that the alkaline CT has a measured pH between 8 and 9, which may be due to the presence of carbonate compounds (Wang et al. 2015a). Therefore, we propose that CT could be used to provide liming amelioration to acidic soils along with other coal wastes (e.g. fly ash) (Ram and Masto 2014). For this purpose, CT might be sold as commercial products (such as soil conditioners) without the need for further processing and manufacturing with other additives. Alternatively, an acidic agent can be added to reduce the pH of the CT to a range of 6–7 for conditioning of all kinds of soil. The option of acidic agents includes but is not limited to the use of lime (Bezdicek et al. 2003; Moir and Moot 2010), elemental sulphur (Liu et al. 2015), iron sulphate (Simiele et al. 2020), and acidic food waste/compost (Sundberg et al. 2013). Lime, sulphur, and compost can also improve the soil’s physical structure and provide nutrients for microorganism growth. For instance, iron sulphate supplement was found to reduce As availability in soil (Simiele et al. 2020).

### Application of coal tailings for improving soil fertility 

Size of soil particles and the carbon content in soil have a direct impact on several soil features, such as water retention capacity (Singh and Verma 2011), nutrient retention capacity (Ersahin et al. 2006), and retention capacity of pesticides and fertilisers (Gaines and Gaines 1994; Farlin et al. 2013). Maintaining these capacities is essential for sustainable agricultural production. In general, these capacities are the strongest in clay, moderate in silt, and the weakest in sand. Nonetheless, sandy soil could improve water drainage properties, which is beneficial for heavy clayey soil. While clayey soils are usually highly susceptible to compaction due to intensive agricultural activities on the land or reduced tillage (Baumgartl and Horn 1991), loamy soil is considered the most suitable soil type for agricultural production. The latter has the right clay–silt–sand mixture (Parikh and James 2012). Our results showed that CT2 has a better soil texture for agricultural applications in comparison with CT1. The former is more suitable for direct field application to provide nutrients or alleviate soil acidity.

The current agricultural practices (e.g., fertilisation, irrigation, mechanical harvesting, pesticide applications) are associated with organic matter decomposition and increased CO_2_ emission (Trost et al. 2013). Organic carbon in soil is an integral component affecting several key soil properties, such as the soil structure, soil fertility, water holding capacity, and erosion resistance (Godde et al. 2016). These properties are generally insufficient for Australian soils as they are highly weathered. Since carbon is the most abundant element in the tailing samples, it can be inferred that the application of CT as a soil conditioner will significantly increase the organic carbon content in the soil. Furthermore, carbonate in CT can provide liming effect, and the Ca supplement can support better soil aggregation and promote soil microbial activities (Holland et al. 2018). In addition, soil microbial activities are correlated with the particle size distribution as increased microbial diversity was found in smaller-sized particles. Several studies indicated that N-fixing bacteria such as *Nitrosospira* and *Nitrosomonas* preferably hosted nitrification on the surface of silt particles (Lowe and Hinds 1983; Catroux and Schnitzer 1987; Hemkemeyer et al. 2018). Therefore, the clay and silt components in CT could be beneficial for promoting soil microbial activities. With large amounts of organic matter and nutrients in CT, there is no doubt that CT can be used for bio-inoculation of beneficial microbiota in poor soil.

## Conclusions

Coal tailings from two Australian mining sites have been evaluated for their potential use in soil amelioration. It is found that the coal tailings are alkaline with loamy or loamy sand textures. The tailings contain a reasonable amount of macronutrients (~ 3% w/w), high carbon (C), and low heavy metals and metalloids (As, Cd, Se, Cu, Zn, and Pb). The tailings have been used as a soil conditioner to treat greenhouse tomato plants. After the treatment, there was an increase in leaf K, Ca, and Mg contents. This study highlights the potential utilisation of coal tailings as a soil conditioner as they contain high levels of carbon, and macro & micronutrients. The high pH of CT may be the factor hindering tomato growth in the high-rate treatment. The coal tailings could be further processed by reducing the pH value and adding other beneficial materials. The improved coal tailings might be used for crops in both the greenhouse and the field. Alternatively, the alkaline coal tailings might be directly used to provide liming amelioration to acidic soils.

In conclusion, the application of CT in agriculture is likely to provide a new avenue towards global sustainable development goals by converting coal waste into soil conditioners that can reduce the environmental impact of coal waste and chemical fertilisers and potentially increase agricultural productivity in the future. However, further research should be conducted to assess the effect of CT on the photosynthetic rate of crops. It should be noted that the tomato plants in the greenhouse failed to produce enough tomato fruits due to insufficient pollination. Further field trials should be conducted to study the effect of coal tailings on the quality and yield of tomato fruits.

## Data Availability

The authors declare that all data supporting the findings of this study are available within the article.

## References

[CR1] Adiansyah JS, Haque N, Rosano M, Biswas W (2017). Application of a life cycle assessment to compare environmental performance in coal mine tailings management. J Environ Manag.

[CR2] Amoah-Antwi C, Kwiatkowska-Malina J, Thornton SF, Fenton O, Malina G, Szara E (2020). Restoration of soil quality using biochar and brown coal waste: a review. Sci Total Environ.

[CR65] Australian Government (2021) Resources and energy quarterly: June 2021. Department of Industry S, Energy and Resources. Office of the Chief Economist

[CR3] Azadi M, Edraki M, Faezeh F, Ahn J (2019). Opportunities for mineral carbonation in Australia’s mining industry. Sustainability.

[CR4] Babla MH, Tissue DT, Cazzonelli CI, Chen Z-H (2020). Effect of high light on canopy-level photosynthesis and leaf mesophyll ion flux in tomato. Planta.

[CR5] Babla M, Katwal U, Yong M-T, Jahandari S, Rahme M, Chen Z-H, Tao Z (2022). Value-added products as soil conditioners for sustainable agriculture. Resour Conserv Recycl.

[CR6] Baumgartl T, Horn R (1991). Effect of aggregate stability on soil compaction. Soil Tillage Res.

[CR7] Bezdicek D, Beaver T, Granatstein D (2003). Subsoil ridge tillage and lime effects on soil microbial activity, soil pH, erosion, and wheat and pea yield in the Pacific Northwest, USA. Soil Tillage Res.

[CR8] Białowiec A, Koziel JA, Manczarski P (2019). Stomatal conductance measurement for toxicity assessment in zero-effluent constructed wetlands: effects of landfill leachate on hydrophytes. Int J Environ Res Public Health.

[CR9] Bojórquez-Quintal E, Escalante-Magaña C, Echevarría-Machado I, Martínez-Estévez M (2017). Aluminum, a friend or foe of higher plants in acid soils. Front Plant Sci.

[CR10] Budak B, Salman A, Khalvati MA (2020). Integration of coal mine tailing and mycorrhizal fungi to associate lolium perenne and poa pratences tensis seed germination and growth period. Fresenius Environ Bull.

[CR11] Carpio MJ, Sánchez-Martín MJ, Rodríguez-Cruz MS, Marín-Benito JM (2021). Effect of organic residues on pesticide behavior in soils: a review of laboratory research. Environments.

[CR12] Catroux G, Schnitzer M (1987). Chemical, spectroscopic, and biological characteristics of the organic matter in particle size fractions separated from an Aquoll. Soil Sci Soc Am J.

[CR13] Chen L, Stehouwer R, Wu M, Kost D, Guo X, Bigham JM, Beeghly J, Dick WA (2013). Minesoil response to reclamation by using a flue gas desulfurization product. Soil Sci Soc Am J.

[CR14] Cheng M, Wang H, Fan J, Xiang Y, Tang Z, Pei S, Zeng H, Zhang C, Dai Y, Li Z (2021). Effects of nitrogen supply on tomato yield, water use efficiency and fruit quality: a global meta-analysis. Sci Hortic.

[CR15] Clark R, Zucker N, Urpelainen J (2020). The future of coal-fired power generation in Southeast Asia. Renew Sustain Energy Rev.

[CR16] Clemente R, Sáez-Tovar JA, Bernal MP (2020). Extractability, distribution among different particle size fractions, and phytotoxicity of Cu and Zn in composts made with the separated solid fraction of pig slurry. Front Sustain Food Syst.

[CR17] Dellantonio A, Fitz WJ, Repmann F, Wenzel WW (2010). Disposal of coal combustion residues in terrestrial systems: contamination and risk management. J Environ Qual.

[CR18] Diao X, Yuan C-G, Wu J, Zhang K, Zhang C, Gui B (2018). Mercury fractions in gypsum and estimation of mercury emission from coal-fired power plants. Fuel.

[CR19] EPA (2007). Method 3051A (SW-846): microwave assisted acid digestion of sediments, sludges, and oils, revision 1.

[CR20] Ersahin S, Gunal H, Kutlu T, Yetgin B, Coban S (2006). Estimating specific surface area and cation exchange capacity in soils using fractal dimension of particle-size distribution. Geoderma.

[CR21] Farlin J, Gallé T, Bayerle M, Pittois D, Braun C, El Khabbaz H, Lallement C, Leopold U, Vanderborght J, Weihermueller L (2013). Using the long-term memory effect of pesticide and metabolite soil residues to estimate field degradation half-life and test leaching predictions. Geoderma.

[CR22] Feng X, Liu W, Dai H, Qiu Y, Zhang G, Chen Z-H, Wu F (2020). HvHOX9, a novel homeobox leucine zipper transcription factor, positively regulates aluminum tolerance in Tibetan wild barley. J Exp Bot.

[CR23] Fu B, Liu G, Mian MM, Sun M, Wu D (2019). Characteristics and speciation of heavy metals in fly ash and FGD gypsum from Chinese coal-fired power plants. Fuel.

[CR24] Gaines T, Gaines S (1994). Soil texture effect on nitrate leaching in soil percolates. Commun Soil Sci Plant Anal.

[CR25] Godde CM, Thorburn PJ, Biggs JS, Meier EA (2016). Understanding the impacts of soil, climate, and farming practices on soil organic carbon sequestration: a simulation study in Australia. Front Plant Sci.

[CR26] Han D, Xu L, Wu Q, Wang S, Duan L, Wen M, Li Z, Tang Y, Li G, Liu K (2021). Potential environmental risk of trace elements in fly ash and gypsum from ultra–low emission coal–fired power plants in China. Sci Total Environ.

[CR27] Hatje V, Pedreira RM, de Rezende CE, Schettini CAF, de Souza GC, Marin DC, Hackspacher PC (2017). The environmental impacts of one of the largest tailing dam failures worldwide. Sci Rep.

[CR28] Haynes R (2009). Reclamation and revegetation of fly ash disposal sites–challenges and research needs. J Environ Manag.

[CR29] Hemkemeyer M, Dohrmann AB, Christensen BT, Tebbe CC (2018). Bacterial preferences for specific soil particle size fractions revealed by community analyses. Front Microbiol.

[CR30] Holland J, Bennett A, Newton A, White P, McKenzie B, George T, Pakeman R, Bailey J, Fornara D, Hayes R (2018). Liming impacts on soils, crops and biodiversity in the UK: a review. Sci Total Environ.

[CR31] Juan L, Jian-Min Z, Zeng-Qiang D, Chang-Wen D, Huo-Yan W (2007). Effect of CO_2_ enrichment on the growth and nutrient uptake of tomato seedlings. Pedosphere.

[CR32] Kanai S, Moghaieb RE, El-Shemy HA, Panigrahi R, Mohapatra PK, Ito J, Nguyen NT, Saneoka H, Fujita K (2011). Potassium deficiency affects water status and photosynthetic rate of the vegetative sink in green house tomato prior to its effects on source activity. Plant Sci.

[CR33] Li W, Chen L, Zhou T, Tang Q, Zhang T (2011). Impact of coal gangue on the level of main trace elements in the shallow groundwater of a mine reclamation area. Min Sci Technol.

[CR34] Liu H, Liu Z (2010). Recycling utilization patterns of coal mining waste in China. Resour Conserv Recycl.

[CR35] Liu X, Zhao Z, Duan B, Hu C, Zhao X, Guo Z (2015). Effect of applied sulphur on the uptake by wheat of selenium applied as selenite. Plant Soil.

[CR36] Liu W, Feng X, Cao F, Wu D, Zhang G, Vincze E, Wang Y, Chen Z-H, Wu F (2021). An ATP binding cassette transporter HvABCB25 confers aluminum detoxification in wild barley. J Hazard Mater.

[CR37] Lowe L, Hinds A (1983). The mineralization of nitrogen and sulphur from particle-size separates of gleysolic soils. Can J Soil Sci.

[CR38] Manoharan V, Yunusa I, Loganathan P, Lawrie R, Skilbeck C, Burchett M, Murray B, Eamus D (2010). Assessments of class F fly ashes for amelioration of soil acidity and their influence on growth and uptake of Mo and Se by canola. Fuel.

[CR39] Mohammadi R, Azadmehr A, Maghsoudi A (2020). Enhancing of competitive adsorptive removal of zinc and manganese from aqueous solution by iron oxide-combusted coal gangue composite. Sep Sci Technol.

[CR66] Moir JL, Moot DJ (2010) Soil pH, exchangeable aluminium and lucerne yield responses to lime in a South Island high country soil. In: Proceedings of the New Zealand Grassland Association. pp 191–196.

[CR40] O’Carrigan A, Babla M, Wang F, Liu X, Mak M, Thomas R, Bellotti B, Chen Z-H (2014). Analysis of gas exchange, stomatal behaviour and micronutrients uncovers dynamic response and adaptation of tomato plants to monochromatic light treatments. Plant Physiol Biochem.

[CR41] Ontl TA, Schulte LA (2012). Soil carbon storage. Nat Educ Knowl.

[CR42] Ouhadi V, Yong R, Shariatmadari N, Saeidijam S, Goodarzi A, Safari-Zanjani M (2010). Impact of carbonate on the efficiency of heavy metal removal from kaolinite soil by the electrokinetic soil remediation method. J Hazard Mater.

[CR43] Parikh SJ, James BR (2012). Soil: the foundation of agriculture. Nat Educ Knowl.

[CR44] Park I, Tabelin CB, Jeon S, Li X, Seno K, Ito M, Hiroyoshi N (2019). A review of recent strategies for acid mine drainage prevention and mine tailings recycling. Chemosphere.

[CR45] Placek A, Grobelak A, Hiller J, Stępień W, Jelonek P, Jaskulak M, Kacprzak M (2017). The role of organic and inorganic amendments in carbon sequestration and immobilization of heavy metals in degraded soils. J Sustain Dev Energy Water Environ Syst.

[CR46] Ram L, Masto R (2014). Fly ash for soil amelioration: a review on the influence of ash blending with inorganic and organic amendments. Earth Sci Rev.

[CR47] Simiele M, Lebrun M, Miard F, Trupiano D, Poupart P, Forestier O, Scippa GS, Bourgerie S, Morabito D (2020). Assisted phytoremediation of a former mine soil using biochar and iron sulphate: effects on As soil immobilization and accumulation in three *Salicaceae* species. Sci Total Environ.

[CR48] Singh S, Verma C (2011). Water retention characteristic from particle size distribution and bulk density data of soils. Proc Indian Natl Sci Acad.

[CR49] Singh S, Parihar P, Singh R, Singh VP, Prasad SM (2016). Heavy metal tolerance in plants: role of transcriptomics, proteomics, metabolomics, and ionomics. Front Plant Sci.

[CR50] Skaggs T, Arya L, Shouse P, Mohanty B (2001). Estimating particle-size distribution from limited soil texture data. Soil Sci Soc Am J.

[CR51] Skłodowski P, Maciejewska A, Kwiatkowska J (2006). The effect of organic matter from brown coal on bioavailability of heavy metals in contaminated soils. Soil and water pollution monitoring, protection and remediation.

[CR67] Spain AV, Tibbett M (2012) Coal mine tailings: development after revegetation with salttolerant tree species. Paper presented at the Mine Closure 2012: Seventh International Conference on Mine Closure, Perth, 2012/09/25

[CR68] Standards Australia (2012) Methods of testing soils for engineering purposes, Method 3.6.1: soil classification tests—determination of the particle size distribution of a soil—standard method of analysis by sieving, AS 1289.3.6.1. Sydney, Australia

[CR69] Standards Australia (2020) Methods of testing soils for engineering purposes, Method 3.6.3: soil classification tests—determination of the particle size distribution of a soil—standard method of fine analysis using an hydrometer, AS 1289.3.6.3. Sydney, Australia

[CR52] Standards Australia (2009). Composts, soil conditioners and mulches, AS 4454.

[CR53] Sundberg C, Yu D, Franke-Whittle I, Kauppi S, Smårs S, Insam H, Romantschuk M, Jönsson H (2013). Effects of pH and microbial composition on odour in food waste composting. Waste Manag.

[CR54] Suzuki M, Umeda H, Matsuo S, Kawasaki Y, Ahn D, Hamamoto H, Iwasaki Y (2015). Effects of relative humidity and nutrient supply on growth and nutrient uptake in greenhouse tomato production. Sci Hortic.

[CR55] Tirez K, Vanhoof C, Hofman S, Deproost P, Swerts M, Salomez J (2014). Estimating the contribution of sampling, sample pretreatment, and analysis in the total uncertainty budget of agricultural soil pH and organic carbon monitoring. Commun Soil Sci Plant Anal.

[CR56] Tremain P, Zanganeh J, Hugo L, Curry S, Moghtaderi B (2014). Characterization of “chailings”: a char created from coal tailings. Energy Fuels.

[CR57] Trost B, Prochnow A, Drastig K, Meyer-Aurich A, Ellmer F, Baumecker M (2013). Irrigation, soil organic carbon and N_2_O emissions. A review. Agron Sustain Dev.

[CR70] U.S. Energy Information Administration (2016) International energy outlook 2016: Chapter 4: Coal. Administration USEI

[CR58] Wang C, Li W, Yang Z, Chen Y, Shao W, Ji J (2015). An invisible soil acidification: Critical role of soil carbonate and its impact on heavy metal bioavailability. Sci Rep.

[CR59] Wang D, Chakraborty S, Weindorf DC, Li B, Sharma A, Paul S, Ali MN (2015). Synthesized use of VisNIR DRS and PXRF for soil characterization: Total carbon and total nitrogen. Geoderma.

[CR60] Wang Q, Wang D, Li Z, Zhang L, Feng X (2021). Mercury in desulfurization gypsum and its dependence on coal properties in coal-fired power plants. Fuel.

[CR61] Ward G (1964). Greenhouse tomato nutrition—a growth analysis study. Plant Soil.

[CR71] World Nuclear Association (2019) Australia’s Electricity (2019). Available via World Nuclear Association. https://www.world-nuclear.org/information-library/country-profiles/countries-a-f/appendices/australia-s-electricity.aspx. Accessed 05 Dec 2021

[CR62] Yunusa IA, Loganathan P, Nissanka S, Manoharan V, Burchett MD, Skilbeck CG, Eamus D (2012). Application of coal fly ash in agriculture: a strategic perspective. Crit Rev Environ Sci Technol.

[CR63] Zhang R, Wang D, Zhang Y, Du T (2018). Effects of green substrates composed of coal gangue on the growth of *Trifolium repens* L. and its resistance to heavy metal pollution. Chin J Appl Environ Biol.

[CR64] Zhou H, Bhattarai R, Li Y, Si B, Dong X, Wang T, Yao Z (2021). Towards sustainable coal industry: turning coal bottom ash into wealth. Sci Total Environ.

